# Does vitamin A supplementation protect schoolchildren from acquiring soil-transmitted helminthiasis? A randomized controlled trial

**DOI:** 10.1186/1756-3305-7-367

**Published:** 2014-08-15

**Authors:** Hesham M Al-Mekhlafi, Tengku Shahrul Anuar, Ebtesam M Al-Zabedi, Mohamed T Al-Maktari, Mohammed AK Mahdy, Abdulhamid Ahmed, Atiya A Sallam, Wan Ariffin Abdullah, Norhayati Moktar, Johari Surin

**Affiliations:** Department of Parasitology, Faculty of Medicine, University of Malaya, 50603 Kuala Lumpur, Malaysia; Department of Medical Parasitology, Faculty of Medicine and Health Sciences, Sana’a University, Sana’a, Yemen; Department of Medical Laboratory Technology, Faculty of Health Sciences, Universiti Teknologi MARA (Puncak Alam Campus), 42300 Kragujevac, Selangor Malaysia; Department of Biochemistry, Faculty of Medicine and Health Sciences, Sana’a University, Sana’a, Yemen; Department of Biology, Faculty of Natural and Applied Sciences, Umaru Musa Yar’adua University, Katsina, Katsina State Nigeria; Faculty of Medicine, SEGi University College, Kota Damansara, Kragujevac, Selangor Malaysia; Department of Pediatrics, Faculty of Medicine, University of Malaya, 50603 Kuala Lumpur, Malaysia; Department of Parasitology and Medical Entomology, Faculty of Medicine, Universiti Kebangsaan Malaysia, Jalan Raja Muda Abdul Aziz, 50300 Kuala Lumpur, Malaysia

**Keywords:** Soil-transmitted helminths, Vitamin A, Randomized clinical trial, Malaysia

## Abstract

**Background:**

Despite the intensive global efforts to control intestinal parasitic infections, the prevalence of soil-transmitted helminth (STH) infections is still very high in many developing countries particularly among children in rural areas.

**Methods:**

A randomized, double-blind, placebo-controlled trial was conducted on 250 Aboriginal schoolchildren in Malaysia to investigate the effects of a single high-dose of vitamin A supplementation (200 000 IU) on STH reinfection. The effect of the supplement was assessed at 3 and 6 months after receiving interventions; after a complete 3-day deworming course of 400 mg/daily of albendazole tablets.

**Results:**

Almost all children (98.6%) were infected with at least one STH species. The overall prevalence of ascariasis, trichuriasis and hookworm infection was 67.8%, 95.5% and 13.4%, respectively. Reinfection rates of *Ascaris*, *Trichuris* and hookworm were high; at 6 months, assessment reached 80% of the prevalence reported before treatment. There were no significant differences in the reinfection rates and intensities of STH between vitamin A supplemented-children and those who received placebo at 3 and 6 months (p *>* 0.05).

**Conclusions:**

Vitamin A supplementation showed no protective effect against STH reinfection and this could be due to the high endemicity of STH in this community. Long-term interventions to reduce poverty will help significantly in reducing this continuing problem and there is no doubt that reducing intestinal parasitic infection would have a positive impact on the health, nutrition and education of these children.

**Trial registration:**

This trial was registered at clinicaltrials.gov as NCT00936091.

## Background

Soil-transmitted helminth (STH) infections, particularly *Ascaris lumbricoides*, *Trichuris trichiura* and hookworm infections are still considered as the most prevalent infections of humankind [1]. These infections together with schistosomiasis represent more than 40% of the disease burden caused by all tropical diseases, excluding malaria [2]. Despite the intermittent deworming programmes conducted by the public and private sectors to control intestinal parasitic infections, the prevalence of STH infections is still very high in many developing countries including Malaysia, particularly among rural and Aboriginal children [1, 3]. This situation is not uncommon in developing countries with newly emerging economies where neglected tropical diseases like STH infections are rife in communities that do not receive effective control programmes and treatment [4].

Mass deworming has proved to be an effective public health intervention that reduces worm burden [5]. In poor communities with high STH endemicity, treated children are frequently reinfected as a result of their continuous exposure to parasites and the prevalence and intensity of infections could be as high as the pre-treatment situation after a period of six months [6, 7]. Hence, there is a need for the development of more cost-effective measures that reduce the disease burden as well as the rates of reinfection. Few previous studies have investigated the effects of trace elements and vitamins on the parasitic infection [8–10]. The World Health Organization (WHO) has recommended that the delivery of vitamin A capsules and anthelmintic tablets should be simultaneous as worm infections and vitamin A deficiency (VAD) have the same geographical distribution [11].

Vitamin A is a fat soluble vitamin that is found in different forms such as retinol and provitamin A carotenoids. It is an essential nutrient for cell proliferation, immune system function and vision [12]. It is also an important nutrient for the gene expression and excretion of growth hormone [13]. Moreover, vitamin A derivatives play a major role in the development of the central nervous system [14]. Besides that, it helps maintain the integrity of skin and mucus membranes that act as a barrier to pathogens [15]. Previous *in vivo* studies have shown beneficial effects for vitamin A supplementation on tissue inflammation and innate and adaptive immunity in humans by downregulating inflammatory responses, enhancing immunoglobulin production, promoting the differentiation of T and B cells and modulating cytokine production [16, 17]. However, very little is known about the effects of vitamin A supplements on STH infections.

The question posed by the present study was whether vitamin A supplementation would alter parasitosis and protect children from acquiring infections of high intensities. We assumed the following possible scenarios a) restoration of normal vitamin A levels could result in low parasitic reinfection rates throughout the overall improvements in health status of the supplemented children, b) low prevalence with low intensity due to decreased parasite establishment and survival as a result of improvement in the host immune response by vitamin A supplementations, and c) a novel mechanism has been recently reported that an increase in the rate of epithelial cell turnover in the intestine acts like an “epithelial escalator” to expel intestinal parasites [18]. This was found to be under immune control by the cytokine interleukin-13. Vitamin A may have a dual effect here: as a key element in the functioning of the immune system and as an essential nutrient for epithelial cell proliferation [12, 19]. Within this context, this study was carried out to investigate whether vitamin A supplementation can protect Malaysian Aboriginal schoolchildren from acquiring or developing STH infections.

## Methods

### Study area and subjects

**Figure 1:**
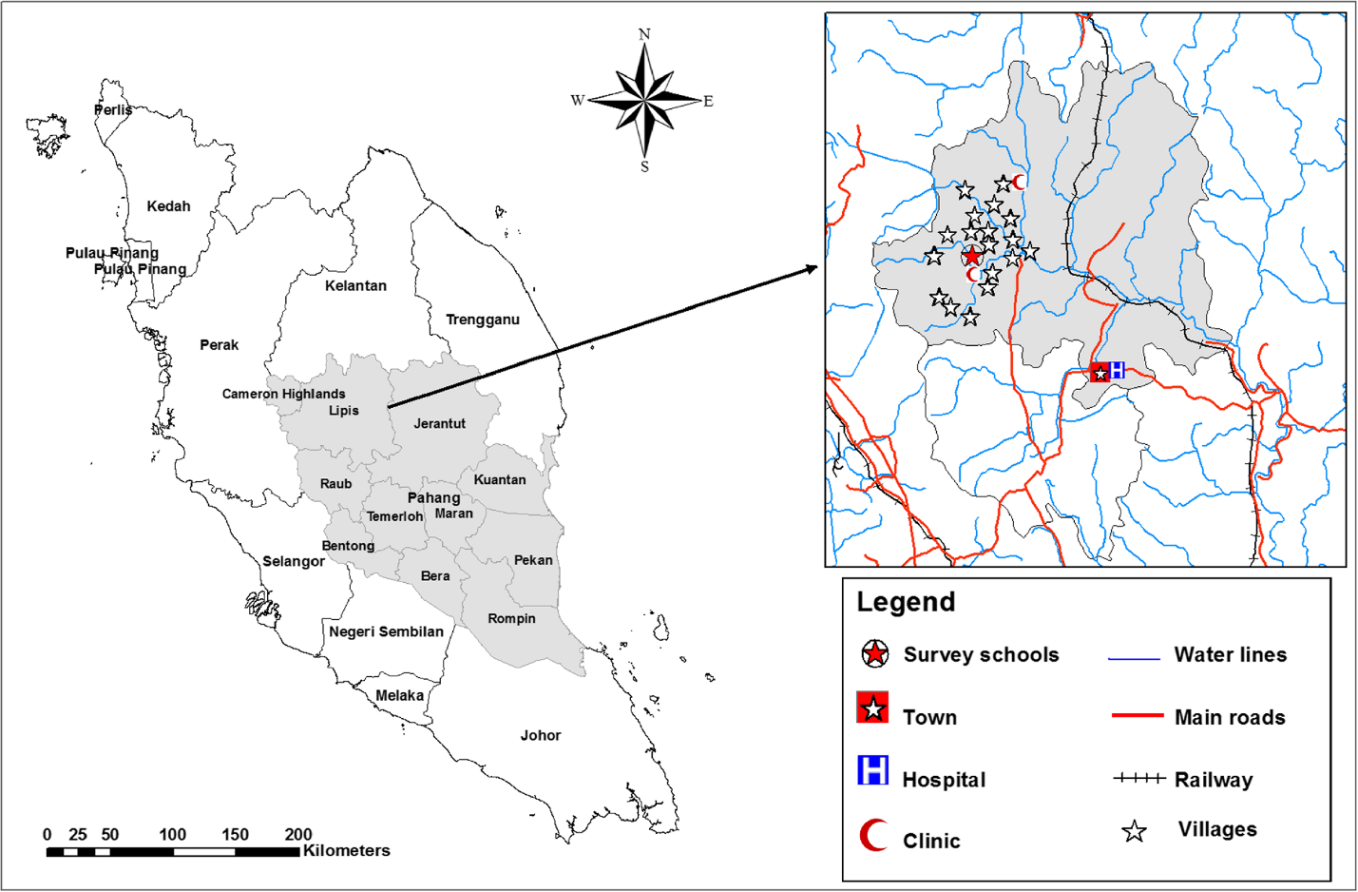
**A geographic map showing the study area (location of school and villages in Lipis district).**

This study was undertaken in Pos Betau, Pahang, Malaysia. Sekolah Kebangsaan Betau (the Betau National School), a primary school for Aboriginal children was selected for this study. This area is considered as a remote area; consisting of 18 villages, it is located in a valley region about 50 km from the town of Kuala Lipis and 200 km northeast of Kuala Lumpur (Figure 1). Most of the residents work as farmers, labourers, rubber tappers and some do other jobs such as selling forest products.

**Figure 2:**
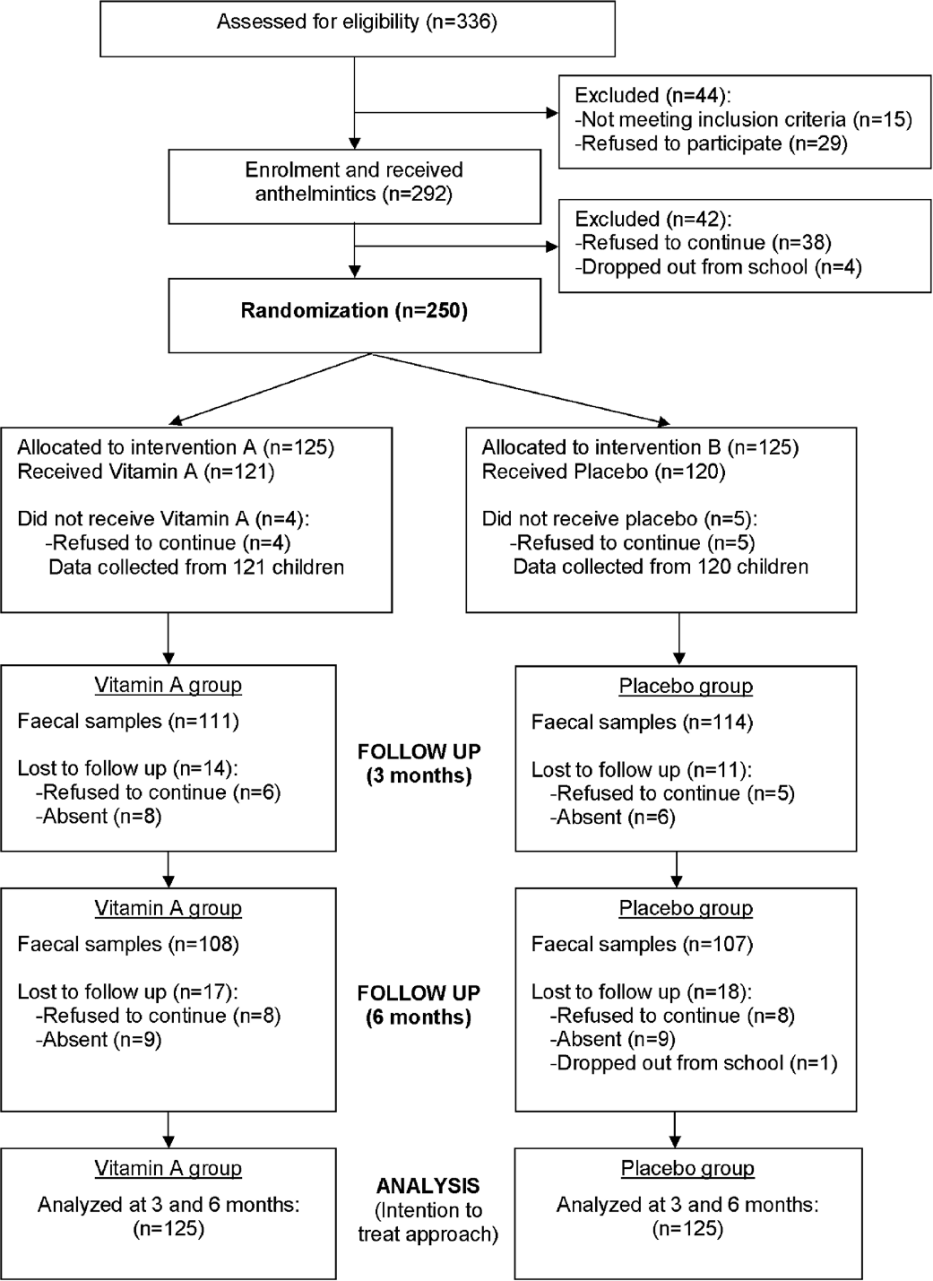
**Flow chart of data collection and follow up.**

A sample size of 214 children (including 20% attrition loss), 107 per intervention arm, was estimated to give the study at least 80% power at 5% significance to detect a 10% or more difference in the prevalence and intensity of parasitic infections between the vitamin A supplemented group and the placebo group. The school had an enrolment of 502 pupils in grades one to six. There were 405 pupils in the target age range of 7–12 years. Of these, 69 were absent at the time of enrolment, 29 refused to participate, and 15 were excluded because they had infections with fever at the time of enrolment. Finally, 292 eligible children received anthelmintics and 250 of them agreed to participate in the intervention part of this study. Data on the socioeconomic status of subjects were also collected using a standard questionnaire. The children were followed up for a period of 6 months as previous studies among Aboriginal children in rural Malaysia revealed that the re-infection rate of STH was high and by 6 months after complete deworming the prevalence and intensity of infections were similar to pre-treatment levels [7, 20]. Descriptions of the trial profile, data collection and follow-up were illustrated according to the CONSORT guideline shown in Figure 2.

### Trial design and interventions

This study was a randomized, double-blind, placebo-controlled trial (Trial Registration: clinicaltrials.gov identifier: NCT00936091). After baseline screening for the eligibility, examination for STH infections, participants received anthelmintics and were re-examined to ensure complete deworming, 250 children were assigned randomly, using a free on-line randomization programme from http://www.randomization.com, into two groups (125 children per group) to receive vitamin A supplements or placebo. The trial was blinded in several ways. For example, the vitamin supplement and placebo capsules were identical in appearance. At the school, neither the person administering the supplements, nor the child receiving the capsule, was aware of the intervention. Faecal samples were coded and the person who processed and examined the specimens were not aware which treatment group any sample corresponded to.

Albendazole tablets (GlaxoSmithKline, London, UK) were used in this study as the anthelmintic treatment and the regime used was a 3-day course of 400 mg/daily. Each white chewable tablet contains 400 mg albendazole as the active ingredient. Tablets were provided in white, well sealed plastic bottles containing 100 tablets each. Each child chewed the tablet before swallowing it with some water, while being observed by a researcher, medical officer, and a teacher (Direct Observed Therapy). The efficacy of the treatment was assessed after 12–14 days and children who were still infected were treated again with a single dose of albendazole 400 mg [6].

Vitamin A supplements and placebos were provided by Tablets (India) Ltd (Chennai, India) and were gelatinous and reddish opaque capsules containing 200,000 IU vitamin A in 200 μL peanut oil with 10 μg vitamin E as a preservative or identical capsules containing the 200 μL peanut oil with 10 μg vitamin E only. The capsules were provided in identical, dark brown and well sealed glass bottles containing 50 capsules each. The content of vitamin A in supplement capsules was confirmed and the capsules were encoded (A and B), the code was kept confidential by personnel who were not involved in the study, when the study ended the code was broken. Each child received the capsule of the relevant group under a direct observed therapy. The dose was followed by two pieces of freshly fried banana (locally known as Pisang Goreng), which is rich in oil that provides a perfect medium for vitamin A absorption and ensures maximum utilization of the capsule’s contents. In addition, these fried bananas are a favourite snack in Malaysia and were readily accepted by the recruited children.

### Ethical consideration

This study was conducted according to the guidelines laid down in the Declaration of Helsinki and all procedures were approved by the Medical Ethics Committee of the University of Malaya Medical Centre, University of Malaya, Malaysia. Due to the high rate of illiteracy among these communities, the committee approved the verbal consent procedures instead of the signed informed consent. During the visits to the school and the villages, community meetings were held with the headmaster of the school, the heads of the villages, the parents and their school-age children before the commencement of the study in order to give a clear explanation of the objectives of the study. By marking in the lists of students’ names, informed verbal consents were obtained from the participants and their parents and the final lists were witnessed by the headmaster and the heads of the villages.

### Parasitology

Fresh faecal samples were collected at baseline, after deworming and at 3 and 6 months after the interventions. The samples were examined by the Kato-Katz and Harada Mori techniques for the presence of STH; *Ascaris lumbricoides*, *Trichuris trichiura* and hookworm eggs [21, 22]. Egg counts, as a measure of worm burden, were also carried out using the Kato-Katz technique and the results were recorded as eggs per gram of stool (epg). Intensity of infection was graded as heavy, moderate, or light according to criteria proposed by the World Health Organization [21]. For *Ascaris*, *Trichuris* and hookworm infections, egg counts of ≥ 50,000 epg, ≥ 10,000 epg and ≥ 4,000 epg, respectively, are regarded as heavy infections while egg counts of < 5,000 epg, > 1,000 epg and > 2,000 epg, respectively, are regarded as light infections.

### Statistical analysis

Statistical analysis of the data was performed using the *Statistical Package for Social Sciences for Windows* SPSS (version 13, 2004). Distribution of quantitative variables were examined for normality using the Shapiro-Wilk test before analysis. Egg counts were found to be not normally distributed, however, there are biological justifications for using the arithmetic mean (±standard deviation, SD) rather than the median or geometric mean to express the egg counts of each STH species [23, 24]. As all infected children were dewormed at baseline, the analysis of STH reinfection was based on the children who were infected at each follow up assessment, adjusted for intention to treat [6].

The effects of the interventions were analyzed by subtracting the increments after treatment in the placebo group from the increments in the supplement group. Chi-square test and Fisher’s exact test were used to compare the reinfection rates between vitamin A and placebo groups while Mann Whitney U test and Wilcoxon signed-rank test were used to compare the intensity of infections (mean epg) between both groups and with related baseline intensity, respectively. In order to avoid bias and violation of power of the study due to missing data after randomization, intention-to-treat analysis was used to analyze the effects of interventions on STH reinfection [25]. The assumption of good or poor outcome by including patients with missing responses in the denominator but not the numerator was implicated when the reinfection rates were calculated [26].

## Results

### General characteristics of subjects and follow-up

A total of 292 Aboriginal schoolchildren participated in this study. Throughout the visits for deworming, 38 (13.0%) children subsequently decided to discontinue and 4 (1.4%) children left the school (dropped out of school). The median age of these children was 10 years (interquartile range 8, 11). After randomization, there were no significant differences in the variables and characteristics between the two groups and this indicated that the randomization process distributed all variables and possible confounders equally between the two groups (Table 1). Data was collected over a period of eight months throughout several visits to the selected school; before interventions (baseline) and at 3 and 6 months after commencement of interventions.

**Table 1:** Baseline characteristics of the schoolchildren involved in the intervention study after randomization*

Characteristics	Vitamin A	Placebo
N	125	125
Male/Female	63/62	63/62
Age (years)^a^	10 (8, 11)	10 (9, 11)
Serum retinol level (μmol/L)^a^	1.12 (0.69, 1.47)	1.14 (0.69, 1.40)
Weight (kg)^b^	22.7 ± 4.5	22.6 ± 5.5
Height (cm)^b^	123.8 ± 7.5	122.6 ± 8.4
Paternal education level (at least 6 years)^c^	38 (30.4)	43 (34.4)
Maternal education level (at least 6 years)^c^	25 (20.0)	28 (22.4)
Low household income (<RM500)^c^	102 (81.6)	98 (78.4)

^a^Median (Interquartile range).

^b^Mean ± SD.

^c^Number (%).

*No significant differences in the variables and characteristics between vitamin A and placebo groups.

Almost 32% of the fathers had formal education of at least 6 years. On the other hand, only 21.2% of the mothers had similar formal education. Poverty is predominant in this community; 80% of the families had a household income below RM500 (US$1 = RM3). Moreover, median retinol concentrations of children in both groups were almost similar. More than half (56.0%) of the children participating in this study had low serum retinol level (i.e. below 1.05 μmol/L).

### Prevalence and distribution of STH

Almost all children (98.6%) were infected either by one or more of the STH species. The overall prevalence of ascariasis, trichuriasis and hookworm infections was 67.8%, 95.5% and 13.4%, respectively. Almost one-third (29.8%) of the children had heavy trichuriasis while 22.3% had heavy ascariasis. All hookworm infections in this population were of light intensity. Almost two thirds (68.2%) of the children had mixed infections; ascariasis and trichuriasis (53.4%) were the most prevalent co-infection while 11.3% of the children were infected with the three species (*A. lumbricoides*, *T. trichiura* and hookworm).

### Effects of vitamin A supplementation on STH reinfection rates

Reinfection rates of *Ascaris*, *Trichuris* and hookworm after 3 and 6 months of interventions are shown in Table 2. Overall, reinfection rates of *A. lumbricoides*, *T. trichiura* and hookworm reached 28.3%, 39.8% and 25.4%, respectively of the pre-treatment levels. Findings at the 3 months assessment showed that there was no significant difference in the reinfection rates between children allocated vitamin A and the placebo (p > 0.05). Likewise, the reinfection rates of *A. lumbricoides*, *T. trichiura* and hookworm reached almost three-quarters, two-thirds and a half of the pre-treatment levels. The differences of reinfection rates between the two groups at this stage were still not significant (p *>* 0.05).

**Table 2:** **Effects of vitamin A supplementation on reinfection rates of STH after 3 and 6 months of the interventions among Aboriginal schoolchildren**

Infections/groups	Baseline	After 3 months ^a^	After 6 months ^a^
***Ascaris***			
Vitamin A	69.4 (60.7, 76.9)	27.8 (17.6, 36.1)	76.8 (61.5, 79.1)
Placebo	65.8 (57.0, 73.7)	28.7 (17.7, 36.8)	73.1 (60.0, 77.5)
*P-value*	0.552	0.781	0.453
***Trichuris***			
Vitamin A	96.7 (91.8, 98.6)	39.9 (29.2, 46.5)	65.8 (53.1, 68.7)
Placebo	97.5 (92.9, 99.1)	39.7 (27.6, 44.7)	66.5 (55.8, 70.1)
*P-value*	0.710	0.983	0.847
**Hookworm**			
Vitamin A	14.9 (9.6, 22.3)	17.4 (4.6, 37.1)	56.4 (30.8, 69.1)
Placebo	10.8 (6.5, 17.7)	33.3 (11.4, 56.4)	51.9 (30.5, 65.0)
*P-value*	0.349	0.485^b^	0.411

All values are prevalence/reinfection rates in percentages (95% CI).

^a^Reinfection rate (95% CI) of infected subjects at 3 or 6 months: (Reinfection rates = Number of infected subjects at 3 or 6 months/number of infected subjects before deworming x 100), Chi-square test.

^b^Fisher’s exact test.

### Effects of vitamin A supplementation on STH reinfection intensities

The effects of the interventions on the intensities of STH reinfection after 3 and 6 months of interventions are shown in Table 3. At 3 months, the intensities of *A. lumbricoides*, *T. trichiura* and hookworm were 32%, 38% and 29% of the intensities at the pre-treatment level, respectively. As a result of deworming, the intensities of STH infections among children of both groups after 3 months of receiving the interventions were significantly different from the baseline intensities (Wilcoxon signed-rank test; p *<* 0.001). The figure became different at 6 months; the intensity of *A. lumbricoides* was almost 86% of the baseline intensity while the intensities of *T. trichiura* and hookworm were 75% and 42%, respectively. The results also revealed that the intensities of *T. trichiura* and hookworm at this stage were significantly different from the baseline in both groups (p *<* 0.05). However, the intensities of *A. lumbricoides* were not significantly different from the intensities at baseline in both groups (p *>* 0.05). Overall, there were no significant differences in the intensities of STH infections over 3 and 6 months between children allocated to the vitamin A and placebo groups (Mann Whitney U test; p *>* 0.05).

**Table 3:** **Effects of vitamin A supplementation on intensities of STH reinfection after 3 and 6 months of the interventions among Aboriginal schoolchildren** *

Infection/Group	Baseline	After 3 months	After 6 months	*P*
***Ascaris***				n.s.
Vitamin A	19,869 (9,744)	896 (263)^a,1^	14,867 (5,246)	
Placebo	16,072 (8,151)	1,339 (519)^a,1^	13,368 (6,367)	
Total	17,978 (8,473)	1,112 (411)^a,1^	14,113 (5,415)	
***Trichuris***				n.s.
Vitamin A	5,474 (2,031)	439 (190)^a,1^	2,859 (799)^a,2^	
Placebo	5,624 (3,449)	722 (240)^a,1^	3,678 (562)^a,2^	
Total	5,548 (3,230)	579 (212)^a,1^	3,270 (622)^a,2^	
**Hookworm**				n.s.
Vitamin A	59 (20)	9 (8)^a,1^	14 (11)^a,2^	
Placebo	57 (19)	6 (7)^a,1^	11 (8)^a,2^	
Total	58 (17)	7 (4)^a,1^	12 (5)^a,2^	

*All values are arithmetic mean counts of egg per gram faeces (SD).

^a^Significant difference from baseline (Wilcoxon signed-rank test): ^1^(*P <* .001), ^2^(*P <* .01).

^P^P-values for the differences between vitamin A and placebo groups (Mann Whitney U test; not significant).

## Discussion

Soil-transmitted helminthiases caused by *Ascaris lumbricoides*, *Trichuris trichiura* and hookworm are among the most common Neglected Tropical Diseases (NTDs) by infecting about 1,200 million people worldwide with the greatest morbidity being among children and mothers of childbearing age [3, 27]. The main impact of STH infections is their association with malnutrition, vitamin A deficiency (VAD) and iron deficiency anaemia (IDA), which may have effects at the community level with regard to work and productivity in adults and growth, learning and school performance in children [28–31]. As recommended by the WHO, vitamin A capsules and deworming tablets are distributed together by control programmes in many countries [11]. However, very little is known about the effects of vitamin A supplements on STH infections. We investigated the effects of vitamin A supplementations (mega-dose; 200,000 IU) on the STH reinfection.

The findings of this study showed that the reinfection rate of STH after three months was high and almost similar in both groups where almost half of the children were reinfected with at least one species of STH. The same situation continued with the findings at 6 months where almost 80% of the children were reinfected. The findings showed that the reinfection rates of STH were similar among the children in both groups suggesting that there was no protective effect for vitamin A supplementations against STH reinfection in these children. This could be due to the very high reinfection rates and the suggested explanation was that continuous exposure to the infective stages of STH was the major determinant of risk of helminth reinfection while nutritional factors were believed to modulate rather than eliminate susceptibility to reinfection. Previous studies revealed an alarmingly high prevalence of STH infections among Aboriginal schoolchildren in rural Malaysia [3, 32].

Similarly, there were no significant differences in the intensities of STH reinfection between the two groups at 3 and 6 months. Findings after 3 months showed that the mean epg of *Ascaris*, *Trichuris* and hookworm were significantly different from the baseline findings. This could be due to biological determinants rather than the effects of the supplements as the placebo group also showed the same differences. Besides endemicity, the deposition of eggs for each female worm per day varies according to the type of worm. It is known that the adult *A. lumbricoides* female worm produces approximately 250,000 eggs per day. A much lower number, 3,000-25,000, is produced by the *T. trichiura* female while the hookworm female produces between 3,000-6,000 in the case of *Necator americanus* and 10,000-30,000 by *Ancylostoma duodenalis* [33]. The egg output of these worms was found to be related to the stage of maturity, age of the worm and number of worms in the gut [34]. This present study showed that the total mean epg of *Ascaris* reinfection after six months was very close to the pre-treatment mean epg (14,113 compared to 17,978) and this figure was higher than in *Trichuris* and hookworm. The higher reinfection rate by six months and higher egg deposition of *Ascaris* could be the possible reason [35]. Two-thirds of these children had mixed infections (co-infections), which may affect the bioavailability of vitamin A throughout the period of study. However, the prevalence of co-infections was similar in both groups as a result of the randomization process. Moreover, the reinfection rates of individual helminth species and co-infections among children were found to be similar in both groups at 3 and 6 months assessments, when adjusted to the co-infection status at baseline.

Comparing these findings with other reports elsewhere, a previous study assessed the benefit of a high-dose of vitamin A supplementation on reinfections with *Ascaris* after deworming among 328 indigenous pre-school children in Panama [36]. Although their study provides evidence that combined vitamin A supplementation and deworming reduces *Ascaris* reinfections in children, the lack of a control group to compare the effects of the supplements limits the interpretation of the results. Moreover, the higher prevalence of mixed infections coupled with the higher intensities of infections reported in our cohort may contribute to the non-significant effects of the supplement. A randomized, double-blind, placebo-controlled trial aimed at evaluating the effect of vitamin A and zinc supplementation on *A. lumbricoides* infections showed that the infections were reduced significantly among children who received combined vitamin A and zinc compared with those who received either vitamin A or zinc alone [37]. This study was conducted among children aged below two years and the prevalence of *Ascaris* (28.0%) was low compared to our study. Moreover, a prospective randomized, double-blind, placebo-controlled showed a significant reduction in the prevalence of new parasitic infections, especially with *Giardia lamblia*, among Brazilian children who received 100,000- 200,000 IU vitamin A at enrollment, 4 and 8 months, and followed for 36 months when compared to those who received placebo [38].

The effects of different micronutrient supplementations on the intestinal parasitic reinfections were investigated. Previous studies found no significant effect for zinc supplementation on the intestinal parasitic reinfections [8, 9]. A positive impact for iron supplementations on helminths reinfection rates was also reported [6]. Adults supplemented with 60 mg elemental iron twice-weekly for 12 months had significantly lower reinfection rates of *A. lumbricoides*, *T. trichiura* and *S. mansoni* compared to adults given placebos. However, iron supplementation had no effect on reinfection intensities in adults. Besides its important role in the functioning of the immune system, iron supplements may result in unfavourable conditions in the host gut that stimulate the parasites expulsion. Oral iron supplementations are given at least 3 days a week and have enough time to produce such unfavourable conditions but this is unlikely with a mega-dose of vitamin A supplementation that is distributed only once every 6 months. While our results showed that vitamin A supplementation does not have an added benefit on STH infections, regular deworming and vitamin A supplementation may have positive impacts on STH infections and VAD, respectively.

## Conclusions

The prevalence and reinfection rates of STH were found to be very high among Aboriginal children in rural Malaysia, undermining the efforts of deworming programmes. Vitamin A supplementation showed no positive impact on STH reinfection rates and intensities and this could be due to the high endemicity of these helminths in this community. Orang Asli children are living in the same conditions and have the same epidemiological characteristics for the STH infections, malnutrition and vitamin A status. Therefore, these findings can be generalized to other Orang Asli children in other states. These findings speak for implementing innovative and integrated measures to control STH infections. Long-term interventions to reduce poverty will help significantly in reducing this continuing problem and there is no doubt that reducing intestinal parasitic infection would have a positive impact on the health, nutrition and education of these children.
